# Morquio‐like dysostosis multiplex presenting with neuronopathic features is a distinct *GLB1*‐related phenotype

**DOI:** 10.1002/jmd2.12211

**Published:** 2021-03-08

**Authors:** Sylvia Stockler‐Ipsiroglu, Nahid Yazdanpanah, Mojgan Yazdanpanah, Marioara Moisa Popurs, Nataliya Yuskiv, Mara Lúcia Schmitz Ferreira Santos, Chong Ae Kim, Carolina Fischinger Moura de Souza, Charles Marques Lourenço, Carlos Eduardo Steiner, Andressa Federhen, Luciana Giugliani, Débora Maria Bastos Pereira, Luz Elena Durán‐Carabali, Roberto Giugliani

**Affiliations:** ^1^ Department of Pediatrics University of British Columbia Vancouver Canada; ^2^ Division of Biochemical Genetics BC Children's Hospital Vancouver Canada; ^3^ BC Children's Hospital Research Institute Vancouver Canada; ^4^ Hospital Infantil Pequeno Príncipe Curitiba Brazil; ^5^ Instituto da Criança, Faculdade de Medicina, USP São Paulo Brazil; ^6^ Serviço de Genética Médica, HCPA Porto Alegre Brazil; ^7^ Faculdade de Medicina Centro Universitário Estácio Ribeirão Preto Brazil; ^8^ Departamento de Genética Médica, Faculdade de Medicina UNICAMP Campinas Brazil; ^9^ Programa de Pós‐Graduação em Ciências Biológicas: Fisiologia, UFRGS Porto Alegre Brazil; ^10^ Departamento de Genética UFRGS Porto Alegre Brazil; ^11^ Programa de Pós‐Graduação em Genética e Biologia Molecular, UFRGS Porto Alegre Brazil; ^12^ INAGEMP Porto Alegre Brazil; ^13^ DR BRASIL, HCPA Porto Alegre Brazil

**Keywords:** dystonia, keratan sulfate, mucopolysaccharidosis type IVB, spondyloepiphyseal dysplasia, type 3 GM1 gangliosidosis, β‐galactosidase

## Abstract

**Background:**

Morquio B disease (MBD) is a distinct *GLB1*‐related dysostosis multiplex presenting a mild phenocopy of *GALNS*‐related Morquio A disease. Previously reported cases from European countries carry the W273L variant on at least one *GLB1* allele and exhibit a pure skeletal phenotype (*pure MBD*). Only a minority of MBD cases have been described with additional neuronopathic findings (*MBD plus*).

**Objectives and Methods:**

With the aim to further describe patterns of MBD‐related dysostosis multiplex, we analyzed clinical, biochemical, and genetic features in 17 cases with *GLB1*‐related dysostosis multiplex living and diagnosed in Brazil.

**Results:**

About 14 of the 17 individuals had three or more skeletal findings characteristic of Morquio syndrome. Two had no additional neuronopathic features (*pure MBD*) and 12 exhibited additional neuronopathic features (*MBD plus*). Three of the 17 cases had mild dysostosis without distinct features of MBD. Seven of the 12 *MBD plus* patients had signs of spinal cord compression (SCC), as a result of progressive spinal vertebral dysostosis. There was an age‐dependent increase in the number of skeletal findings and in the severity of growth impairment. *GLB1* mutation analysis was completed in 10 of the 14 *MBD* patients. T500A occurred in compound heterozygosity in 8 of the 19 alleles.

**Conclusion:**

Our study extends the phenotypic spectrum of GLB1‐related conditions by describing a cohort of patients with MBD and GM1‐gangliosidosis (*MBD plus*). Targeting the progressive nature of the skeletal manifestations in the development of new therapies for GLB1‐related conditions is warranted.

## 1 INTRODUCTION

Morquio B disease (MBD), also called mucopolysaccharidosis IVB (OMIM # 253010), is a rare lysosomal storage disorder, which is allelic to GM1‐gangliosidosis (OMIM # 230500). Both MBD and GM1‐gangliosidosis are caused by variants in *GLB1*, the structural gene coding for lysosomal acid ß‐galactosidase. β‐galactosidase facilitates the degradation of complex carbohydrates bound to a variety of structurally unrelated molecules such as gangliosides, proteoglycans, and N‐ and O‐linked glycoproteins.^1^

MBD is characterized by a distinct type of dysostosis multiplex initially described as Morquio syndrome.^2^, ^3^ Typical skeletal features of Morquio syndrome include short stature with a disproportionally short trunk, kyphoscoliosis, pigeon chest (pectus carinatum), short neck, large appearing head with midface hypoplasia and mandibular protrusion, large appearing joints (elbows, wrists, knees, ankles), coxa and genua valga, flat feet, and joint laxity. Characteristic radiological findings include platyspondyly and vertebral beaking, odontoid hypoplasia, spinal canal narrowing, hip dysplasia, dysplasia of the carpal and tarsal bones as well as shortening and epi‐and metaphyseal dysplasia of long bones (eg, shortening of the ulna and sloping of the distal ends of radius and ulna). Corneal clouding, cardiac valve disease, and tracheal stenosis are additional findings found in the Morquio phenotype.

Currently, two genetic conditions are known to cause Morquio‐like dysostosis multiplex: *GALNS*‐related Morquio A disease (OMIM 25300) and *GLB1*‐related MBD. Biochemically, both conditions are characterized by an accumulation of keratan sulfate, which is mainly synthesized in the cartilage.^1^, ^4^ However, while *GALNS*‐related Morquio A and *GLB1*‐related MBD share similar patterns of dysostosis multiplex, skeletal manifestations are usually milder in MBD when compared to typical Morquio‐A disease. Along with this notion, we previously could show that the height of adult MBD patients is significantly less compromised than that of those with typical Morquio A disease.^5^

GM1‐gangliosidosis is a neuronopathic disorder with variable onset and severity of neurodegeneration. Depending on age of onset and severity of neurologic manifestations, GM1 gangliosidosis is divided into type 1 (infantile) GM1‐gangliosidosis (OMIM 230500) which begins before age 1 year with hepatosplenomegaly, progressive loss of neurodevelopmental abilities and vision, cherry red macula spot, seizures and dystonia/spasticity; type 2 (late infantile/juvenile) GM1‐gangliosidosis, which is characterized by a later onset of motor and cognitive regression; and type 3 (adult) GM1‐gangliosidosis, which causes extrapyramidal signs (dystonia, ataxia, dysarthria), cardiomyopathy, and variable degrees of intellectual disability.^6^ Variable patterns of visceral manifestations including organomegaly, cardiac valve involvement, and corneal clouding can occur in both MBD and GM1 gangliosidosis. Mild skeletal involvement can be part of GM1 phenotypes, and milder forms of neuronopathic involvement have been reported in MBD^6^, ^7^ .

Biochemically, patterns and distribution of the accumulating substrates across the various tissues and organs are determined by the impact of the underlying GLB mutation on the molecular pathophysiology of the *β*‐galactosidase protein^8^. While accumulation of GM1‐gangliosides in the brain seems most responsible for neurologic manifestations in GM1‐gangliosidosis, keratan sulfate accumulation has been shown in MBD and type 1 (infantile) GM1‐gangliosidosis.^1^

In a recent review of 51 MBD cases,^7^ the majority manifested with progressive growth impairment, kyphoscoliosis, coxa/genua valga, joint laxity, platyspondyly and odontoid hypoplasia, all in the absence of neuronopathic manifestations. Only a minority had additional neuronopathic manifestations such as developmental/intellectual/speech disability, spasticity, ataxia and dystonia in addition to their skeletal features. The term *pure MBD* and *MBD plus* (neuronopathic features) was coined to distinguish both phenotypes.^7^ In this study, W273L was invariably associated with *pure MBD*, both in homo‐ and heterozygosity, but no *GLB1* variant could be identified that was invariably associated with MBD and neuronopathic manifestations.^7^

Most patients with *pure MBD* and the W273L variant have been diagnosed in Europe, but limited knowledge about MBD‐related phenotypes and genotypes is available from patients diagnosed in other continents. There is also limited knowledge about the pattern and extent of the Morquio‐specific dysostosis multiplex in cases with *MBD plus* and how it overlaps with mild GLB1‐related dysostosis.

The aim of this study was to describe the clinical and genetic findings in a cohort of patients living and diagnosed in Brazil, South America's largest country presenting with *GLB1* deficiency and dysostosis multiplex. We were particularly interested in patterns of MBD‐related dysostosis multiplex and their association with neurologic and neurocognitive features of GM1 gangliosidosis.

## 2 METHODS

We performed a retrospective chart review of patients, referred by services from different parts of Brazil, and diagnosed with *GLB1* deficiency at the Medical Genetics Service, Hospital de Clínicas, Porto Alegre, Brazil. The patients presented with dysostosis multiplex with or without additional neurologic/neurocognitive problems. The study was approved by the local ethics review boards of the University of British Columbia as part of a multicenter study on the natural history of GLB1‐reated MBD, and of Ethics Research Committee of Hospital de Clínicas de Porto Alegre (HCPA). The study was funded by the Priest Family Fund for Morquio‐B research, a UBC based steward ship grant.

For data collection, a REDCap database was created comprising the following domains: demographic patient data, disease history, clinical data, biochemical/molecular genetics test results, laboratory research data, and site identification. The digital REDCap database has been hosted by the Women & Children's Health Research Institute (WCHRI)'s Clinical Informatics Core (CRIC) in Edmonton, Alberta as part of the NeuroDevNet/Kid's Brain Health Network research initiatives (www.neurodevnet.ca). The study files were encrypted and study‐related electronic data were stored both on the REDCap database and on a secure password‐protected, limited‐accessed computer at BC Children's hospital for data analysis. Every participant was provided with a study ID and no personal identifiers were collected, apart from e‐mail addresses. The information of each individual was collected by clinical geneticists and included in the REDCap database starting from October 2015 to May 2017.

Dysostosis multiplex was classified as MBD if there were three or more radiological/clinical findings characteristic of the Morquio type of dysostosis multiplex such as platyspondyly and vertebral beaking involving all segments of the spine, odontoid hypoplasia, epi‐and metaphyseal dysplasia of long bones, genua/coxa valga, hip dysplasia, joint laxity/hyperextensible joints, barrel chest/pectus carinatum and short stature (≤third centile, ≤− 2 SD).

Neurologic manifestations were divided into primary and secondary. Primary neurologic manifestations were considered when they were most likely caused by GM1‐related CNS pathophysiology such as extrapyramidal symptoms (ataxia, dystonia, dysarthria, dysphagia, chorea, athetosis), abnormal signal intensity in the basal ganglia and/or white matter, cortical/cerebellar atrophy, and ventriculomegaly. Epilepsy, developmental delay, intellectual disability, progressive loss of cognitive abilities/dementia were also considered as primarily caused by GM1‐related manifestations. Secondary neurologic manifestations where considered when there was clinical and/or radiologic evidence of spinal cord compression (SCC) caused by spinal vertebral stenosis and deformities.

In line with previous recommendations for sub‐classification of MBD,^7^ patients were categorized as *pure MBD* (MBD‐like dysostosis in the absence of neuronopathic manifestations) and *MBD plus* (MBD plus neuronopathic manifestations). Patients who had less than three skeletal manifestations were categorized as mild dysostosis without distinct features of MBD.

## 3 RESULTS

We describe 17 individuals diagnosed with *GLB1* deficiency presenting with various degrees and combinations of dysostosis multiplex and neurologic/neurocognitive dysfunction. Ten of the 17 patients (P1, 2,7,8,9,10,12, 15,16,17) were described previously in the context of clinical findings in adult GM1 gangliosidosis.^9^

There was a preponderance of male patients (13/17). The vast majority of patients had a Portuguese/Iberian ethnic background (11/13). Most of them had attended a special aid school and were relying on caregivers (40%, 73.3%, respectively).

About 14 of the 17 patients had three or more radiological/clinical findings characteristic of the Morquio type of dysostosis multiplex and thus met the criteria for *GLB1‐related MBD* (P 2‐14, P16). About 12 of these 14 patients had additional neuronopathic features (P2, P3, P4, P5, P6, P7, P8, P9, P10, P12, P14, P16) (*MBD plus*), while two had no additional neuronopathic features (P11, P13) (*pure MBD*). Three of the 17 patients (P1, P15, P17) had mild dysostosis without distinct features of MBD. The age of onset was 1 to 7 years of age (median 3.5) for the 12 patients with *MBD plus*, 9 and 10 years for the two patients with *pure MBD* and 0.5, 2, and 4 years for the three patients with mild dysostosis without distinct features of MBD.

**FIGURE 1:**
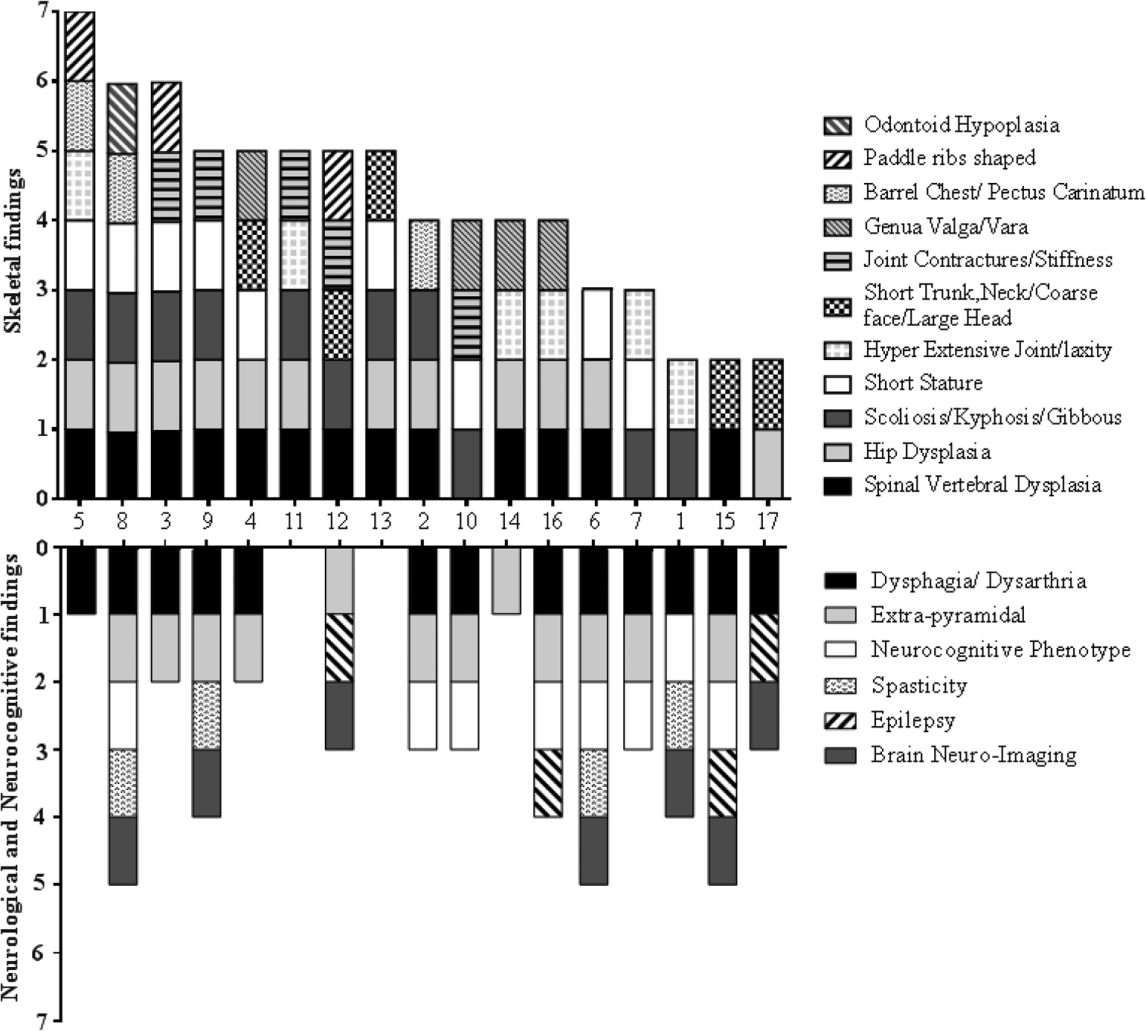
Numbers and types of skeletal and neurological findings in 17 patients with GLB1‐related Morquio‐like dysostosis multiplex

Skeletal and neurologic/neurocognitive features for all 17 patients are shown in Figure 1.

### 3.1 Skeletal features

Among the 14 patients with *GLB1‐related MBD*, the most frequently observed skeletal manifestations were located in the hips and in the spine, with spinal vertebral dysplasia in 12 of the 14, hip dysplasia in 11 of the 14 patients, and scoliosis/ kyphosis/ gibbous in 7 of the 14 patients. Barrel chest/pectus carinatum, hyperextensible joints, and genua valga/vara were found in four (P2, P5, P6, P8), five (P5, P7, P11, P16), and four (P4, P10, P14, P16) *MBD* patients, respectively. Odontoid hypoplasia was reported in only one patient (P8). Patients were from 3 to 34 years (median 19) at the time of the most recent assessment. The number of skeletal manifestations ranged from 3 to 7. Older patients (adults and adolescents) tended to have more skeletal manifestations compared to children.

### 3.2 Growth

Data on age‐related body height were available in nine of the 14 *MBD* patients with seven of them having significant growth restriction (body height < 3%ile/<−2 SD) (Table 1 ).

**TABLE 1:** Skeletal manifestations, growth impairment and neuronopathic manifestations in 17 patients with GLB1‐related dysostosis multiplex

Patient/gender	Phenotype	Age at aassessment (y)	Age at onset (y)	Skeletal features	Neuronopathic features	Body height SD	Body height cm
P5 (m)	*MBD plus*	23	NA	7	1	−3.4	152
P8 (m)	*MBD plus*	34	NA	7	5	−4.25	146
P3 (m)	*MBD plus*	18	7	6	2	−4.55	141
P9 (m)	*MBD plus*	33	3	5	4	−3.0	155^a^
P4 (f)	*MBD plus*	19	5	5	2	−2.8	145
P12 (m)	*MBD plus*	12	5	5	3	NA	NA
P16 (m)	*MBD plus*	3	3	4	4	NA	NA
P2 (m)	*MBD plus*	27	7	4	3	−1.8	127^b^
P10 (f)	*MBD plus*	19	2	4	3	−1.4	150
P14 (m)	*MBD plus*	12	6	4	1	NA	NA
P7 (m)	*MBD plus*	10	4	3	3	−2.6	122
P6 (f)	*MBD plus*	32	3	3	5	“very short stature”	
P11 (m)	*Pure MBD*	16	10	5	0	NA	NA
P13 (m)	*Pure MBD*	21	9	5	0	NA	NA
P15 (m)	*GM1 + D*	4	2	2	5	NA	NA
P1 (m)	*GM1 + D*	9	4	2	4	+0.56	137
P17 (f)	*GM1 + D*	1	0.5	2	3	NA	NA

*Note:* Height: https://simulconsult.com/resources/measurement.html?type=weight.

Abbreviations: *D*, GLB1‐related mild dysostosis without distinct features of MBD; f, female; m, male; NA = not available; SD, standard deviation;

^a^ At 19 years of age.

^b^ At 9 years of age.

### 3.3 Neurologic features

All 12 patients with *MBD plus* had some kind of extrapyramidal movement disorder with the majority having dystonia (n = 9: P 2‐4, 6, 8‐10, 12, 16), and dysarthria/dysphagia (n = 10). Isolated gait ataxia was reported in two patients (P7, P14). Pyramidal movement disorder (spasticity) was found only in combination with dystonia (n = 3: P 8,9,6). Six of the 12 patients with MBD plus had cognitive delay and two had epilepsy.

### 3.4 Brain imaging (MRI/CT)

Information was available from seven patients (P1, 6, 8, 9, 12, 15, 17) and showed abnormal results in all of them. Findings included various types and locations of brain atrophy (general, cerebral, cerebellar, brainstem, ventriculomegaly); enlarged Virchow spaces; central, and abnormal signal intensity and/or atrophy of basal ganglia. Central, subcortical periventricular, or cerebellar dysmyelination/hypomyelination was exclusively observed in these three patients with *mild GLB1‐related dysostosis*.

*Spinal cord compression* (*SCC*) is a secondary neurologic manifestation due to spinal vertebral dysostosis and was reported in six patients with *MBD plus* (P3, P4, P6, P7, P8, P9) and one patient with *pure MBD* (P11). Patients (P6, P8, P9) presented with hyperreflexia, spasticity (ankle clonus), and upper and/or lower limb weakness. Additional bladder incontinence, another characteristic finding in spinal cord compression, was observed in one individual (P11). The clinical diagnosis was backed up by radiological (X‐ray, CT, MRI) evidence of spinal canal narrowing and diffuse reduction of the vertebral bodies in four patients (P8, P11, P13, P14).

#### 3.4.1 Other manifestations

Patients with *GLB1‐related MBD* exhibited the following visceral manifestations: Corneal clouding (P4, P5, P16), cardiac valve pathology (P12, P16), cardiomyopathy (P16), and hepatosplenomegaly (P11). Patients with *GLB1‐related mild dysostosis* had corneal clouding (P17) and hepatosplenomegaly (P15, P17). Cherry red spot, another characteristic finding in GM1 gangliosidosis, was not reported at all.

#### 3.4.2 Biochemical and molecular genetic findings

Values of *β‐galactosidase activity* (measured in leukocytes or fibroblasts using artificial 4‐methylumbeliferyl β‐galactosidase) were available in 15 of the 17 patients. Residual activities ranged between 0.8% and 17.9% of lower limit of normal reference range (Table 2). There was no recognizable pattern allowing an association between type and severity of clinical manifestations and residual β‐galactosidase activity.

**TABLE 2:** Clinical classification, genotype and biochemical findings in 17 patients with *GLB1*‐related dysostosis multiplex

		Genetic		Biochemical			
Patient (gender)	Clinical Classification	Genotype	Nucleotide change	β‐Gal Activity nmol/h/mg (% normal)		Total GAG^a^	Keratansulfate
5 (m)^b^	MBD plus	T500A/NI	c.1498A > G/	10^e^	(12.8%)^g^	NL	—
8 (m)^c^	MBD plus	T500A/R59H	c.1498A > G/c.176G > A	6^e^	(7.7%)	NL	—
3 (m)	MBD plus	T500A/I354S	c.1498A > G/c.1061 T > G	11^e^	(14.1%)	—	Abnormal^h^
9 (m)	MBD plus	T500A/Trp527Leufs*5	c.1498A > G/c.1622–27 insG	14^e^	(17.9%)	—	—
4 (f)	MBD plus	T500A/I354S	c.1061 T > G/c.1498A > G	18^f^	(4.6%)	—	—
12 (m)	MBD plus	—	—	7^e^	(8.9%)		—
16 (m)	MBD plus	—	—	—		—	—
2 (m)	MBD plus	R59H/R201H	c.176G > A/c.602G > A	4^e^	(5.1%)	NL	Abnormal^h^
10 (f)	MBD plus	Thr283GInfs*21/Y64C	C.846delC/C.191A > G	—	—	—	—
14 (m)	MBD plus	T500A/Trp527Leufs*5	c.1498A > G/c.1622‐27 insG	6.9^e^	(8.8%)	126^a^	—
7 (m)	MBD plus	—	—	11^e^	(14.1%)	—	—
6 (f)^d^	MBD plus	T500A/T384S	c.1498A > G/c.1150A > T	15^f^	(3.8%)	—	Abnormal^i^
11 (f)	Pure MBD	T500A/Trp527Leufs*5	c.1498A > G/c.1622‐27 insG	9^e^	(11.5%)	98^a^	—
13 (m)	Pure MBD	—	—	9^e^	(11.5%)	NL	—
15 (m)	GM1 + D	—	—	4.3^e^	(5.5%)	NL	—
1 m	GM1 + D	—	—	3^e^	(3.8%)	NL	—
17 (m)	GM1 + D	—	—	3^f^	(0.8%)	—	—

Abbreviations: GM1 + D, GLB1‐related mild dysostosis without distinct features of MBD; NI, not identified; NL, normal; UD, unspecific dysostosis; —, not available.

^a^ Normal age‐related range: 26‐97 μg GAG/mg creatinine.

^b^ Already published by Kannebley et al. (11)

^c^ Already published by Kannebley et al. (11)

^d^ Already published by Kannebley et al. (11)

^e^ β‐Galactosidase reference range in fibroblasts: 394 to 1440 nmol/h/mg protein.

^f^ β‐Galactosidase reference range in leukocytes: 78‐280  nmol/h/mg protein.

^g^ Percentage (%) of β‐Galactosidase activity compared to lower limit of the reference range.

^h^ Keratan sulfate.

^i^ Chondroitin sulfate/keratan sulfate.

Results of total *glycosaminoglycan* (*GAG) urinary excretion* were available in eight patients. Two patients (P11 with *pure MBD* and P14 with *MBD plus*) had an increased GAG excretion. Three *MBD plus* patients (P2, P3, P6) had an abnormal excretion of keratan sulfate upon fractional analysis of urinary GAGs. Abnormal patterns on urinary oligosaccharides were not reported in any patient.

*GLB1 mutation analysis* was completed in 10 of the 17 patients including one *pure MBD* (P11) and nine *MBD plus* patients. Ninteen alleles were reported. T500A (c.1498A > G) was the most prevalent variant occurring in 8 of the 19 alleles (42%), followed by Trp527Leufs*5 (c.1622‐27insG) (3/19 alleles), I354S (2/19 alleles), and R59H (2/19 alleles). All variants occurred in compound heterozygosity.

## 4 DISCUSSION

We investigated 17 patients with GLB1‐related dysostosis multiplex, 14 of whom were categorized as *GLB1‐related MBD*, and 3 as *GLB1‐related mild dysostosis without distinct features of MBD*.

The main finding in the patients exhibiting the MBD phenotype was a high frequency of neuronopathic manifestations. About 12 of the 14 *MBD* patients had neuronopathic manifestations (*MBD plus*) in addition to their skeletal findings, while only two of these 14 patients had *pure MBD*. The progressive nature of the skeletal disease has been shown in previous cases^7^ and is further supported by the cases reported here who exhibited an increasing number of skeletal findings and an age‐dependent increasing severity of growth impairment. The high rate of SCC found in our *MBD* cases deserves particular attention: seven patients (41%) showed clinical and/or radiological signs of SCC. This finding is in contrast with the 51 *MBD* patients reported previously,^7^ out of whom only four had spinal canal narrowing without and with myelo‐compression. SCC occurs as a consequence of progressive vertebro‐spinal deformity and spinal canal narrowing, causing progressive limb weakness and spasticity as well as bladder and sphincter incontinence. Regular monitoring for this complication is important to provide surgical intervention prior to irreversible damage of the spinal cord.

The preponderance of *MBD plus* in the Latin American patient population studied here is in contrast to the *MBD* cases published recently in which the majority (41/51) had *pure MBD*, while only a minority was found to have *MBD plus*.^7^ One explanation for the discrepancy between both studies resides in the different ethnic and genetic backgrounds of the populations examined: Among the previously reported patients roughly 70% of patients were of Central, South and South Eastern European origin, with W273L being the most prevalent *GLB1* variant. T500A was almost exclusively present in a smaller proportion (25%) of South Western (Iberian, Portuguese) origin, which is ethnically closer to the population studied here.^7^ T500A is a missense mutation located in the β‐domain of the *GLB1* gene. The impact of this variant on β‐galactosidase function (premature degradation, catalytic) has not been fully clarified. Overall data from this and previous studies suggest that among the MBD variants, W273L is the most prevalent variant in Central and Southern Europe, whereas T500A is prevalent in the Iberian/Portuguese and Latin American descendent population.

In the patients analysed here, T500A occurred in compound heterozygosity along with a variety of variants, which have been observed in the neuronopathic forms (type 1, 2, and 3) of GM1 gangliosidosis (Trp527Leufs*5, I354S, T384S, and R59H),^10^, ^11^, ^12^, ^13^, ^14^, ^15^ explaining the neuronopathic element of their clinical phenotypes. Of note, R59H homozygosity has been associated with type 1 GM1 gangliosidosis.^16^ T500A has not yet been reported in homozygosity making a definite genotype‐phenotype correlation possible.

Among the two *MBD plus* cases who do not present T500A in their genotype (P2 [R59H/R201H] and P10 [Y64C/ Thr283GInfs*21]), R210H and Y64C are most likely driving the MBD phenotype. Both variants have previously been observed in late onset (type 3) GM1 gangliosidosis, whereas the respective second alleles (R59H and Thr283GInfs*21) are known to be associated with infantile (type 1) GM1 gangliosidosis.

While the W273L variant has been shown to affect mainly the degradation of keratan sulfate^8^ due to defective ligand recognition,^17^ no such data are known for T500A. Among the patients reported here, keratan sulfate was determined and found to be elevated in only three individuals with two of them carrying the T550A variant on one allele.

Analytical challenges imposed by the use of traditional methods may explain why in our cases keratan sulfate was either not determined or information was mostly restricted to its presence or absence. Quantitative measurements of keratan sulfate using LC‐MS/MS‐based technologies have only recently become available and applied in patients with Morquio‐specific dysostosis multiplex.^18^, ^19^, ^20^ Further studies in MBD patients carrying the T500A variant are needed to determine whether and to what extent this variant is specifically associated with keratan sulfate accumulation.

Several limitations need to be considered in the interpretation of our results. Due to the retrospective data collection, incomplete and missing data are inherent. This is particularly true for genotypic data, which were available only in 10 of the 17 patients. Our registry‐based data did not contain data on growth for all patients. Likewise, the uncertainty about completeness of other clinical and biochemical data limits information about long‐term skeletal and neurologic manifestations as well as about correlations between genotypes and biochemical features.

Significant progress is being made in the development of treatments for GLB1‐related conditions, including chaperone and substrate reduction therapies^21^ and gene therapy.^22^ Knowledge of the natural history and the genotype‐phenotype correlation of *GLB1*‐related conditions associated with the various types of dysostosis multiplex will be essential to inform the design and choice of outcomes in future clinical trials. For this purpose, a patient reported registry, focusing on MBD has been developed by our team^5^ and a database for longitudinal clinical monitoring of patients with GLB1‐related dysostosis and biobanking of biological samples is currently being implemented. Standardized methodologies for the description of the biochemical phenotype such as β‐galactosidase activity and keratan sulfate accumulation, the measurement of thus far poorly described clinical features such as joint laxity and pain, as well as exploratory outcomes such as pro‐inflammatory and collagen‐related factors^23^ should be developed in support of future natural history and therapeutic outcome studies. Extensive studies performed for MPS4A^4^ will serve as valuable templates.

## CONFLICT OF INTEREST

The authors disclose no conflicts of interested related to this paper.

## AUTHOR CONTRIBUTIONS

Sylvia Stockler‐Ipsiroglu and Roberto Giugliani conceived and designed this study and took full responsibility for the paper. Sylvia Stockler‐Ipsiroglu wrote main parts of the article; Mojgan Yazdanpanah and Mojgan Yazdanpanah contributed equally in analysing and illustrating the RedCap data and contributed to writing this article; Marioara Moisa Popurs created and the Redcap database and along with Nataliya Yuskiv coordinated the study at the Candian site; Mara Lúcia Schmitz Ferreira Santos, Chong Ae Kim, Carolina Fischinger Moura de Souza, Charles Marques Lourenço and Carlos Eduardo Steiner contributed with the data collection and analyses; Andressa Federhen, Luciana Giuglian, and Débora Maria Bastos Pereira collected specific information and performed data review; Luz Elena Durán‐Carabali was the coordinator of the study at the Brazilian site. All authors have read and agreed to the final version of the manuscript.
